# A Rapid LC-MS/MS-PRM Assay for Serologic Quantification of Sialylated O-HPX Glycoforms in Patients with Liver Fibrosis

**DOI:** 10.3390/molecules27072213

**Published:** 2022-03-29

**Authors:** Aswini Panigrahi, Julius Benicky, Renhuizi Wei, Jaeil Ahn, Radoslav Goldman, Miloslav Sanda

**Affiliations:** 1 Lombardi Comprehensive Cancer Center, Department of Oncology, Georgetown University, Washington, DC 20057, USA; ap1824@georgetown.edu (A.P.); jb2304@georgetown.edu (J.B.); rw799@georgetown.edu (R.W.); rg26@georgetown.edu (R.G.); 2 Clinical and Translational Glycoscience Research Center, Georgetown University Medical Center, Georgetown University, Washington, DC 20057, USA; 3 Department of Biostatistics, Bioinformatics and Biomathematics, Georgetown University, Washington, DC 20057, USA; ja1030@georgetown.edu; 4 Department of Biochemistry and Molecular & Cellular Biology, Georgetown University, Washington, DC 20057, USA; 5 Max-Planck-Institut fuer Herz-und Lungenforschung, Ludwigstrasse 43, 61231 Bad Nauheim, Germany

**Keywords:** microflow LC-MS, mLC-MS/MS, liver fibrosis, hemopexin, biomarker

## Abstract

Development of high throughput robust methods is a prerequisite for a successful clinical use of LC-MS/MS assays. In earlier studies, we reported that nLC-MS/MS measurement of the O-glycoforms of HPX is an indicator of liver fibrosis. In this study, we show that a microflow LC-MS/MS method using a single column setup for capture of the analytes, desalting, fast gradient elution, and on-line mass spectrometry measurements, is robust, substantially faster, and even more sensitive than our nLC setup. We demonstrate applicability of the workflow on the quantification of the O-HPX glycoforms in unfractionated serum samples of control and liver disease patients. The assay requires microliter volumes of serum samples, and the platform is amenable to one hundred sample injections per day, providing a valuable tool for biomarker validation and screening studies.

## 1. Introduction

Biomarker studies rely heavily on nano-flow liquid chromatography tandem mass spectrometry (nLC-MS/MS) for both the discovery shotgun proteomics and the targeted follow-up validation studies. In contrast to the small molecule analyte quantification, where standard HPLC flow rates for LC-MS analysis are common, the nLC-MS/MS has been favored for peptide quantification primarily because of the sensitivity of analyte detection. However, nLC-MS methods remain technically challenging, time consuming, and less robust [1], which limits their use in clinical laboratories or their applications to large sample sets. 

More recently, researchers have begun to explore capillary columns with a bore wider than the conventional 75 µM ID nano-flow analytical columns [2,3,4]. This allows execution of the LC step of proteomic studies at a microflow rate, and at a substantially higher throughput. The increased flow rate reduces the gradient time and increases the reproducibility and robustness of the measurements [5]. However, in a conventional single spray-tip setup, the higher flow rate diminishes ionization efficiency and lowers sensitivity of detection below acceptable limits for the majority of the peptides in complex samples. This has been addressed by the development of a multi-nozzle emitter that splits the flow evenly into multiple smaller streams, which has been shown to enhance substantially the ionization efficiency [6]. In combination with advances in the sensitivity of the mass spectrometers, the microflow LC-MS/MS (mLC-MS/MS) methods reach sensitivity of detection comparable to that of nLC-MS/MS. Shotgun proteomics studies using mLC-MS/MS have reported identification of close to 10,000 proteins in cell digests, and stability and reproducibility over thousands of runs [5,7]. In these studies, the robustness of the method in high-throughput bottom-up proteomic analyses has been demonstrated using complex cell, tissue, and body fluid digests. The microflow method enabled avoidance of column overloading, resulting in good peak shapes. This, in addition to negligible carryovers, is critical for accurate quantification of compounds by the LC-MS/MS analyses. The method has been adapted for protein biomarker studies using data independent analysis (DIA), parallel reaction monitoring (PRM), and multiple reaction monitoring (MRM) [3,8,9,10]. However, we are not aware of any reports of the use of the mLC-MS/MS for the analysis of O-glycopeptides. 

In this study, we developed a mLC-MS/MS-PRM assay for the quantification of site-specific mucin-type O-glycoforms of hemopexin, which we previously reported as a promising candidate biomarker for the serologic monitoring of liver fibrosis [11,12]. We have shown that the sialylated O-glycoforms of hemopexin (HPX) in serum of patients are associated with advancing fibrosis in hepatitis C-associated liver disease [11]. This may prove useful in the monitoring of the fibrotic liver disease, which affects a large segment of the world’s population, and whose progression can be mitigated by timely lifestyle changes and interventions [13,14]. Our newly optimized method allows for capture of the analytes, desalting, and gradient elution using a one-column setup, directly in a tryptic digest of unfractionated serum, which significantly reduces the time needed for sample preparation and analysis. We used the method to quantify the HPX glycoforms in serum samples of HCV-induced liver disease, and we demonstrate that the mLC-MS/MS-PRM assay offers substantially higher throughput compared to our reported workflow [11], maintains higher sensitivity of detection, and offers a high-throughput serologic assay (100 injections/day) for an improved screening of these glycopeptide biomarker candidates.

## 2. Results and Discussion

Liver biopsy has been the gold standard in the diagnosis of fibrotic changes associated with chronic liver diseases, and non-invasive methods such as liver imaging, ultrasound elastography, and serologic monitoring provide additional options [13]. Serum protein biomarkers, including glycosylation pattern of liver secreted proteins, represent an attractive strategy for serologic monitoring of liver disease (reviewed in [15,16]). We have characterized O-glycoforms of HPX by mass spectrometry [11,12,17] and demonstrated that the relative abundance of the di- and mono-sialylated O-glycoforms increase significantly with the progressing fibrotic liver disease of HCV etiology [11]. Building upon our earlier studies, we aimed to develop a fast mLC-MS/MS assay to quantify the HPX glycoforms at high throughput.

### 2.1. Microflow LC-MS/MS for the Quantification of O-HPX 

We optimized a microflow (1.5 µL/min) LC-MS/MS workflow with 5× higher throughput compared to the earlier nanoflow (0.3 µL/min) method. In a conventional metal/glass needle emitter setup this would translate to a loss of sensitivity because of the dilution of analytes. To circumvent this, we used a multi-nozzle emitter (8-nozzle, Newomics) [6], which has been reported to achieve sensitivity close to routine nLC-MS/MS applications. 

The sample trapping and desalting was achieved within 2 min at a 5 µL/min flow rate using a 20 mm C18 trap column, followed by elution of the analytes at a 1.5 µL flow rate in 3 min, column washing for 2 min, followed by a 6 min equilibration step (total 13 min; for a schematic see Supplementary Figure S1). The time gap between each sample run is negligible, thus making the analysis of approximately 100 samples per day feasible. The analytes were measured by a scheduled PRM assay using an Orbitrap Fusion Lumos Mass Spectrometer (Thermo Scientific, Dreieich, Germany).

**Figure 1:**
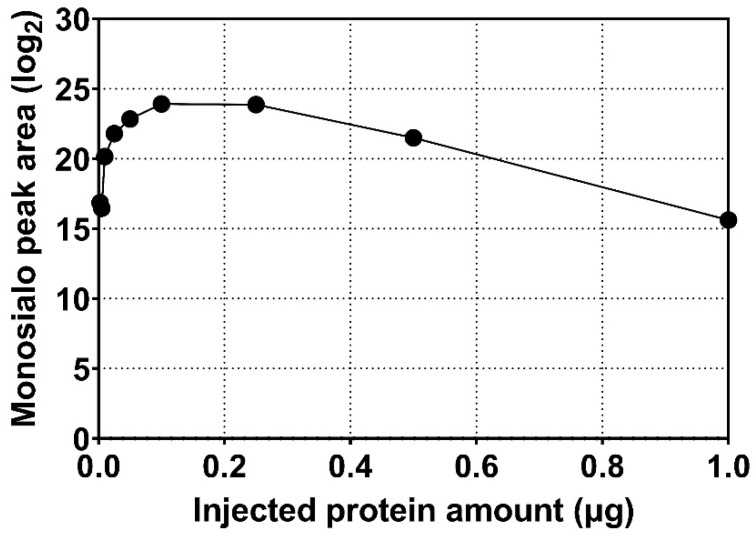
Peak area of tryptic monosialylated O-HPX glycopeptide in relation to the amount of serum protein analyzed by mass spectrometry.

**Figure 2:**
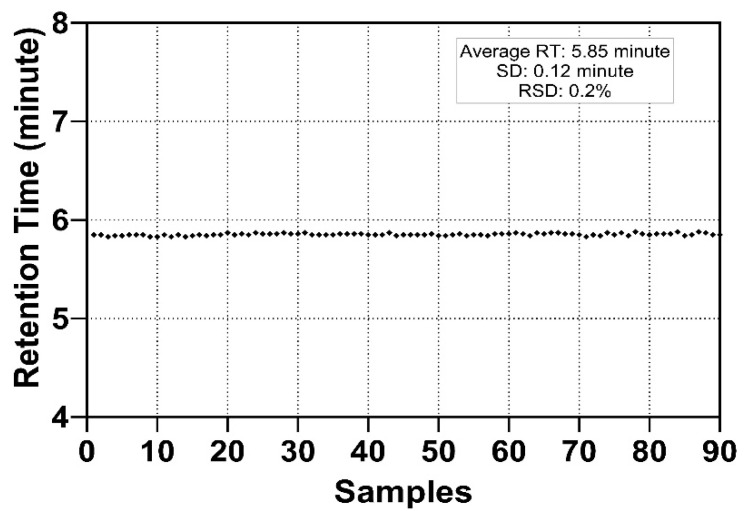
Retention time of a tryptic HPX O-glycopeptide on an Acclaim PepMap 100 C18 column. The consistent elution time at 5.85 ± 0.12 min demonstrates excellent reproducibility.

**Figure 3:**
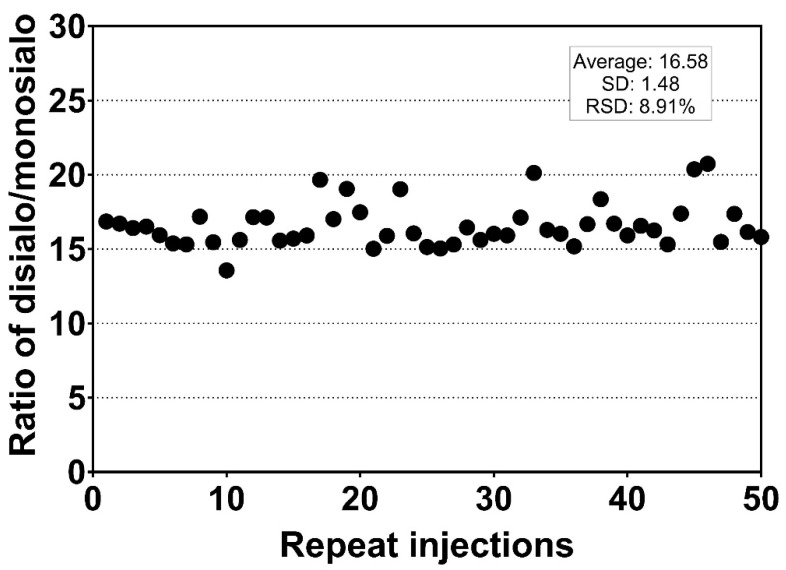
Repeat measurement of S-HPX from one sample by mass spectrometry. Each dot represents the ratio of the *m/z* 916.4 to the *m/z* 843.6 in one injection.

Measurement using serially diluted samples showed optimal sensitivity between 0.1 and 0.2 µg of injected serum protein sample (Figure 1). The retention time (RT) of the analytes was highly reproducible (RSD 0.20%, Figure 2) which is suitable for automated results processing. The S-HPX measurement (i.e., the ratio of disialo *m/z* 916.4/monosialo *m/z* 843.6 analyte) [11] was shown to be consistent over 50 injections (RSD 8.91%, Figure 3), demonstrating outstanding technical reproducibility of the label-free tandem mass spectrometry assay. 

### 2.2. Application of the Micro-Flow LC-MS/MS Assay to Serum Samples of Liver Disease Patients 

**Figure 4:**
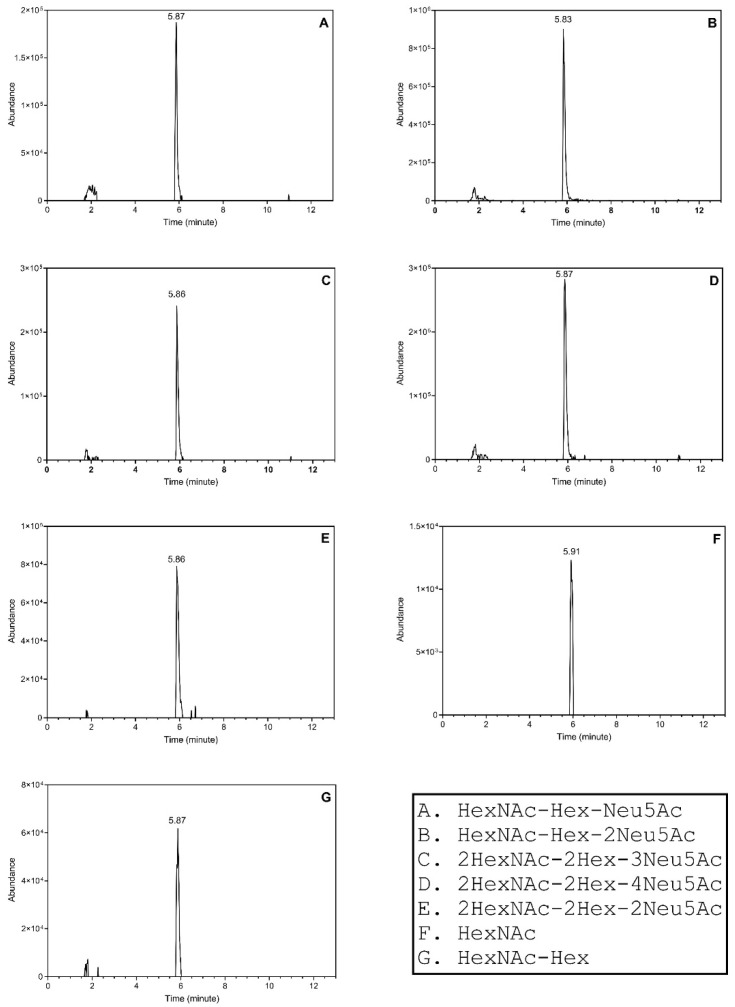
The extracted ion chromatograms showing the elution of the sialylated O-HPX peptides. The composition of the analytes is provided in the bottom right panel.

**Figure 5:**
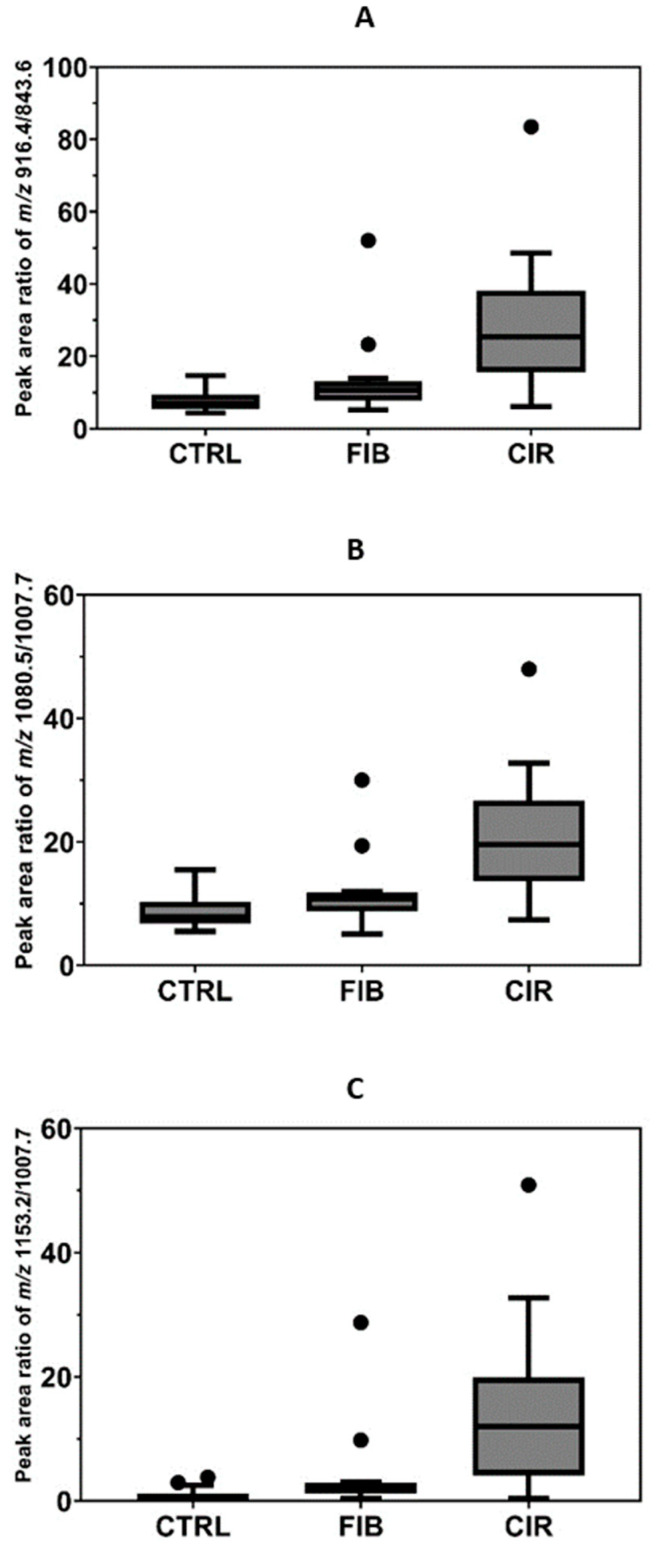
Quantification of S-HPX in control (CTRL, *n* = 15) and progressing stages of liver disease, liver fibrosis (FIB, *n* = 15), and cirrhosis (CIR, *n* = 15). S-HPX, the ratio of monosialylated glycopeptide of the same structure (disialoT/monosialoT) increases significantly (*p* < 0.01) from the control, to the fibrosis and cirrhosis groups. Ratio of (**A**) HexNAc-Hex-2Neu5Ac/HexNAc-Hex-Neu5Ac, (**B**) 2HexNAc-2Hex-3Neu5Ac/2HexNAc-2Hex-2Neu5Ac, (**C**) 2HexNAc-2Hex-4Neu5Ac/2HexNAc-2Hex-2Neu5Ac.

We reported detectability of other O-glycoforms of HPX, including the Tn-antigen, in our previous study; however, we were not able to quantify these analytes in the patient samples [11]. In our current assay, we quantify the additional analytes because of enhanced sensitivity of the current setup in spite of the introduction of faster flow rates (Supplementary Table S1). The inclusion list consisted of multiple O-glycoforms of the N-terminal HPX tryptic peptide [HexNAc (*m/z* 973.5), HexNAc-Hex-Neu5Ac (*m/z* 843.6), HexNAc-Hex-2Neu5Ac (*m/z* 916.4), 2HexNAc-2Hex-2Neu5Ac (*m/z* 1007.7), 2HexNAc-2Hex-3Neu5Ac (*m/z* 1080.5), 2HexNAc-2Hex-4Neu5Ac (*m/z* 1153.2), HexNAc-Hex (*m/z* 770.9)]. Their elution profile shows that the analytes elute within a short window of 5.83–5.91 min (Figure 4). The enhanced detection of the O-HPX glycoforms in unfractionated serum samples using this microflow method may be due to the combination of sample loading capacity and excellent peak shape (Figure 5) obtained at the higher flow rate. With the assumption that minor ionization differences of the glycoforms do not affect the overall results, we calculated the ratios of multiple sialylated to respective monosialylated glycoforms. The ratios of the sialylated O-HPX analytes (S-HPX) were calculated based on the peak areas of the multiple sialylated structures to singly sialylated structures 916.4/843.6, 1080.5/1007.7, and 1153.2/1007.7 using the transitions described previously [11]. 

As a proof of applicability, we quantified S-HPX in serum samples of 15 HCV fibrotic and 15 HCV cirrhotic patients (HALT-C trial participants), and compared the quantities to 15 serum samples of healthy controls. The measurement was undertaken using a fixed volume of serum samples and the measure is normalized by the ratio of the glycoforms of the same protein, as described previously [11]. Statistical analyses were performed to find the association between the different analytes and the disease status. The mean ratio and standard error of 916.4/843.6 in control, fibrotic, and cirrhotic groups was 7.905 ± 0.8562, 13.69 ± 2.942, and 29.99 ± 4.950; and that of 1080.5/1007.7 was 8.802 ± 0.8, 11.65 ± 1.558, and 21.59 ± 2.587; and that of 1153.2/1007.7 was 1.07 ± 1.131, 4.261 ± 1.979, and 14.65 ± 3.49 respectively. One-way ANOVA analysis showed that the relative ratios for the three analytes, 916.4/843.6 (*p* < 0.0001), 1080.5/1007.7 (*p* < 0.0001), and 1153.2/1007.7 (*p* = 0.0004) vary significantly between the control, fibrosis, and cirrhosis groups (Figure 5). Thus, this study expands the number of meaningful analytes for the detection of liver fibrosis. It confirms the results observed in our earlier study, that the S-HPX increases progressively in fibrotic and cirrhotic participants compared to disease-free controls (Figure 5). Further studies are needed to understand the mechanism and biological processes controlling this outcome. Nevertheless, our results show that the mLC–MS/MS-PRM assay has adequate analytical performance for direct quantification of the clinically relevant S-HPX analyte in serum samples. 

Overall, we demonstrate the utility of a 13 min mLC-MS/MS-PRM assay for the quantification of the S-HPX glycoforms diagnostic of liver fibrosis of HCV etiology. The assay is more sensitive compared to that of our earlier report, highly reproducible, and amenable to 100 sample injections per day. Target analyte carryover between the sample injections is negligible (results not shown). In conjunction with a simple sample preparation method without an off-line desalting step, our workflow enables analysis of at least 30 samples per day in triplicate, including necessary QC injections. These parameters would be applicable in a clinical setting. A further increase in the throughput is feasible using a wider-bore capillary column with a higher flow rate, thereby reducing the gradient run time. A multi-nozzle emitter suitable for a flow rate up to 40 µL is commercially available and would support such adjustments. Optimization of a high-flow high-sensitivity methodology would be a focus for future studies. 

## 3. Materials and Methods

### 3.1. Materials

Ammonium bicarbonate, DL-dithiothreitol (DTT), iodoacetamide (IAA) (Sigma-Aldrich St. Louis, MO, USA); sequencing grade trypsin (Promega, Madison, WI, USA)). LC/MS grade Water, 0.1% formic acid in Acetonitrile, 0.1% formic acid in Water (Thermo Fisher Scientific, Waltham, MA, USA). Acclaim PepMap 100 column (Thermo Fisher Scientific, Waltham, MA, USA). 

### 3.2. Sample Processing

Serum samples were processed by trypsin digestion, without any enrichment step, as described earlier [11]. Briefly, 2 µL of each serum sample was diluted to 140 µL with 25 mM ammonium bi-carbonate; the proteins were reduced by 5 mM DTT at 60 °C for 1 h, followed by alkylation with 15 mM iodoacetamide for 20 min at RT in the dark. Residual iodoacetamide was reduced with 5 mM DTT for 20 min at RT. The proteins (20 µL by volume from above) were digested with mass spectrometry grade trypsin (1 µg) at 37 °C O/N. Tryptic peptides were analyzed without further processing to ensure reliable quantification of the glycoforms.

### 3.3. Micro-Flow LC-MS/MS-PRM 

LC-MS/MS analysis was performed using an Ultimate 3000 RSLCnano chromatograph and Orbitrap Fusion Lumos Mass Spectrometer platform (Thermo) with a multi-nozzle emitter (NEWOMICS, Berkeley, CA, USA) used as the microflow sprayer. Glycopeptide separation was achieved in microflow mode using an Acclaim PepMap 100 capillary column 75 μm ID × 20 mm length, packed with C18 5 μm, 300 Å (Thermo). Glycopeptides were separated as follows: starting condition flow 5 µL, 2% ACN, 0.1% formic acid; 0–1 min flow 5 µL, 2% ACN, 0.1% formic acid; 1–2 min flow 1.5 µL, 2–5% ACN, 0.1% formic acid; 2–5 min flow 1.5 µL, 5–98% ACN, 0.1% formic acid; 7–9 min flow 1.5 µL, 98% ACN, 0.1% formic acid; followed by equilibration to starting conditions for an additional 4 min (Supplementary Figure S1). 

We used a Parallel Reaction Monitoring (PRM) workflow with one MS **^1^** full scan (400–1800 *m/z*, resolution 120 K, max IT 50 ms) and scheduled MS/MS fragmentation (Isolation window *m/z* 2.0, HCD fragmentation, resolution 30 K, scan range 200–1400, RF Lens 55%) for the analysis of the sialylated O-HPX glycopeptide TPLPPTSAHGNVAEGETKPDPVTER (Table 1). 

### 3.4. Study Population

Serum samples of participants in the HALT-C trial were obtained from the central repository at the National Institute of Diabetes and Digestive and Kidney Diseases (NIDDK) as described previously [12]. In this study, O-HPX glycoforms comparison was performed in 30 participants (15 HCV fibrotic and 15 HCV cirrhotic patients) and 15 disease-free controls that donated blood samples at Georgetown University (GU) in line with approved IRB protocols. Briefly, the HALT-C trial is a prospective randomized controlled trial of 1050 patients that evaluated the effect of long-term low-dose peginterferon alpha-2a in patients who failed initial anti-HCV therapy with interferon [18]. Liver disease status of the study participants was classified based on biopsy-evaluation into groups of fibrosis (Ishak score 3–4) or cirrhosis (Ishak score 5–6). The two groups of liver disease samples, and the controls, were frequency matched on age, gender, and race (Supplementary Table S2). 

### 3.5. Data Analysis

LC-MS/MS data were processed by Quant Browser (Thermo) with manual confirmation/integration. Peak areas were used for peptide and glycopeptide quantification and data normalization. A specific Y-ion (e.g., loss of whole glycan) was used for the quantification of the O-glycopeptides. The specific backbone fragments (y-ions) were used for the confirmation of the correct O-glycopeptides signal. The details of the MS/MS transitions used for the quantification of each glycoforms are listed in Table 1. Relative intensity of multiple sialylated analyte was calculated by normalizing its peak area to the peak area of monosialylated glycopeptide of the same structure (DisialoT/monosialoT, etc.), as described previously [11].

Statistical analysis for the HCV dataset was performed using GraphPad Prism software (v9.3.1). The ratio of three HPX-sialylated analytes 916.4, 1080.5, and 1153.2, to their respective non-sialylated forms (843.6, 1007.7, and 1007.7), was used as the quantitative measure for evaluation of the liver disease. The mean, standard error of mean, and the one-way ANOVA test was performed to determine the correlation between different analytes and disease status, and the data was visualized by nested Tukey plot.

## Figures and Tables

**Table 1:** Targeted PRM analysis of tryptic O-glycopeptide of HPX: analyte composition, MS data collection parameters, and transitions used for quantitation is highlighted.

Compound	*m/z*	z	Collision Energy (%)	Transitions Used for Quantitation
HexNAc-Hex-Neu5Ac	843.6	4	20	905.8
HexNAc-Hex-2Neu5Ac	916.4	4	20	905.8
2HexNAc-2Hex-3Neu5Ac	1080.5	4	20	905.8
2HexNAc-2Hex-4Neu5Ac	1153.2	4	20	905.8
2HexNAc-2Hex-2Neu5Ac	1007.7	4	20	905.8
HexNAc	973.5	3	20	905.8
HexNAc-Hex	770.9	4	20	905.8

## Data Availability

The datasets generated during the current study are available from the corresponding author on reasonable request.

## Supplementary Materials

The following are available online at https://www.mdpi.com/article/10.3390/molecules27072213/s1, Figure S1: A schematic showing gradient conditions for LC-MS/MS analysis, Table S1: Basic information on samples analyzed in this study, participant demography and disease conditions are provided.

## Sample Availability

Samples of the compounds are available from the authors.

## References

[1] Angel T.E., Aryal U.K., Hengel S.M., Baker E.S., Kelly R.T., Robinson E.W., Smith R.D. (2012). Mass Spectrometry-Based Proteomics: Existing Capabilities and Future Directions. Chem. Soc. Rev..

[2] Vowinckel J., Zelezniak A., Bruderer R., Mülleder M., Reiter L., Ralser M. (2018). Cost-Effective Generation of Precise Label-Free Quantitative Proteomes in High-Throughput by MicroLC and Data-Independent Acquisition. Sci. Rep..

[3] Sun R., Hunter C., Chen C., Ge W., Morrice N., Liang S., Zhu T., Yuan C., Ruan G., Zhang Q. (2020). Accelerated Protein Biomarker Discovery from FFPE Tissue Samples Using Single-Shot, Short Gradient Microflow SWATH MS. J. Proteome Res..

[4] Bian Y., Zheng R., Bayer F.P., Wong C., Chang Y.C., Meng C., Zolg D.P., Reinecke M., Zecha J., Wiechmann S. (2020). Robust, Reproducible and Quantitative Analysis of Thousands of Proteomes by Micro-Flow LC-MS/MS. Nat. Commun..

[5] Kuster B., Bian Y., Bayer F.P., Chang Y.C., Meng C., Hoefer S., Deng N., Zheng R., Boychenko O. (2021). Robust Microflow LC-MS/MS for Proteome Analysis: 38 000 Runs and Counting. Anal. Chem..

[6] Chen Y., Mao P., Wang D. (2018). Quantitation of Intact Proteins in Human Plasma Using Top-Down Parallel Reaction Monitoring-MS. Anal. Chem..

[7] Bian Y., The M., Giansanti P., Mergner J., Zheng R., Wilhelm M., Boychenko A., Kuster B. (2021). Identification of 7000–9000 Proteins from Cell Lines and Tissues by Single-Shot Microflow LC-MS/MS. Anal. Chem..

[8] Bringans S., Ito J., Casey T., Thomas S., Peters K., Crossett B., Coleman O., Ebhardt H.A., Pennington S.R., Lipscombe R. (2020). A Robust Multiplex Immunoaffinity Mass Spectrometry Assay (PromarkerD) for Clinical Prediction of Diabetic Kidney Disease. Clin. Proteom..

[9] Ni W., Jagust W., Wang D. (2021). Multiplex Mass Spectrometry Analysis of Amyloid Proteins in Human Plasma for Alzheimer’s Disease Diagnosis. J. Proteome Res..

[10] Ibrahim S., Lan C., Chabot C., Mitsa G., Buchanan M., Aguilar-Mahecha A., Elchebly M., Poetz O., Spatz A., Basik M. (2021). Precise Quantitation of PTEN by Immuno-MRM: A Tool To Resolve the Breast Cancer Biomarker Controversy. Anal. Chem..

[11] Sanda M., Benicky J., Wu J., Wang Y., Makambi K., Ahn J., Smith C.I., Zhao P., Zhang L., Goldman R. (2016). Increased Sialylation of Site Specific O-Glycoforms of Hemopexin in Liver Disease. Clin. Proteom..

[12] Benicky J., Sanda M., Pompach P., Wu J., Goldman R. (2014). Quantification of Fucosylated Hemopexin and Complement Factor H in Plasma of Patients with Liver Disease. Anal. Chem..

[13] Ginès P., Krag A., Abraldes J.G., Solà E., Fabrellas N., Kamath P.S. (2021). Liver Cirrhosis. Lancet.

[14] El-Serag H.B., Rudolph K.L. (2007). Hepatocellular Carcinoma: Epidemiology and Molecular Carcinogenesis. Gastroenterology.

[15] Mehta A., Herrera H., Block T. (2015). Glycosylation and Liver Cancer. Adv. Cancer Res..

[16] Zhu J., Warner E., Parikh N.D., Lubman D.M. (2019). Glycoproteomic Markers of Hepatocellular Carcinoma-Mass Spectrometry Based Approaches. Mass Spectrom. Rev..

[17] Ma J., Sanda M., Wei R., Zhang L., Goldman R. (2018). Quantitative Analysis of Core Fucosylation of Serum Proteins in Liver Diseases by LC-MS-MRM. J. Proteom..

[18] Di Bisceglie A.M., Shiffman M.L., Everson G.T., Lindsay K.L., Everhart J.E., Wright E.C., Lee W.M., Lok A.S., Bonkovsky H.L., Morgan T.R. (2008). Prolonged Therapy of Advanced Chronic Hepatitis C with Low-Dose Peginterferon. N. Engl. J. Med..

