# Supplementary material for: A Rapid LC-MS/MS-PRM Assay for Serologic Quantification of Sialylated O-HPX Glycoforms in Patients with Liver Fibrosis
Source: Molecules. 2022 Mar 29;27(7):2213. doi: 10.3390/molecules27072213 (PMC9000230; doi:10.3390/molecules27072213)
Supplement: Supplementary file 1 [file molecules-27-02213-s001.zip › molecules-1630773-supplementary.pdf]

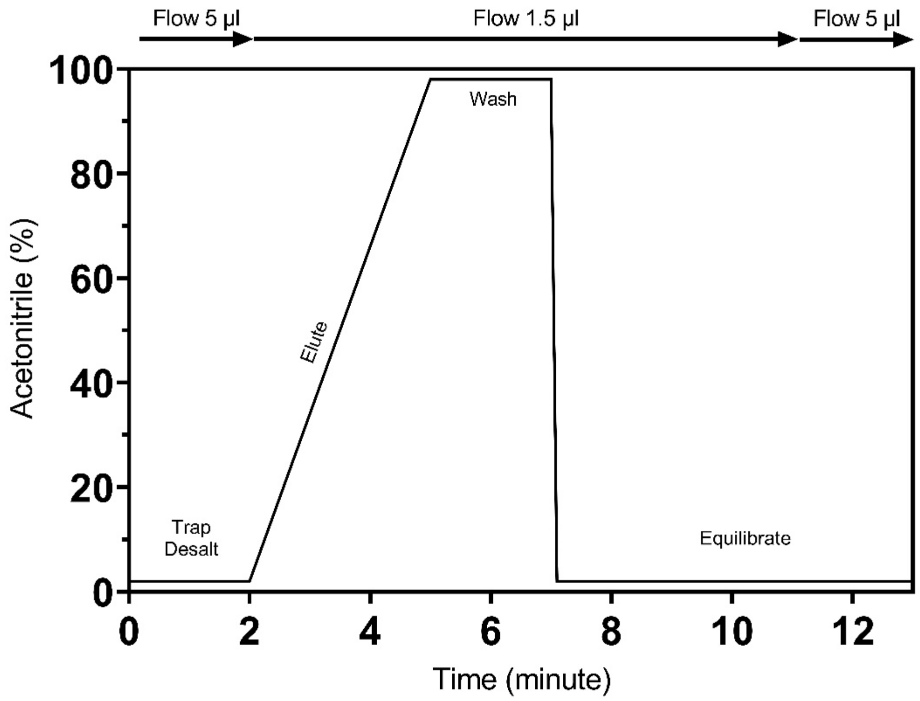

Figure S1. A schematic showing gradient conditions for LC-MS/MS analysis.

Table S1. Comparison of S-HPX measurement using nLC-MS-MRM and mLC-MS-PRM in unfractionated human serum samples. While seven target compounds were identified by mLC method, only two of the target compounds (tick marked) were identified by nLC method.

Methodology	HexNAc	HexNAc-Hex	HexNAc-Hex-NeuAc	HexNAc-Hex-2NeuAc	2HexNAc-2Hex-2NeuAc	2HexNAc-2Hex-3NeuAc	2HexNAc-2Hex-4NeuAc
nLC-MS-MRM	×	×	✓	✓	×	×	×
mLC-MS-PRM	✓	✓	✓	✓	✓	✓	✓

Table S2. Basic information on samples analyzed in this study.

ID	Disease Group	FE-MALE	Age	Race	FI-BRO_ISHAK_S00	FI-BRO_ISHAK_M24	FI-BRO_ISHAK_M48
34916	CIR	0	52	1	6	5	5
35018	CIR	0	51	1	5	5	6
35027	CIR	0	56	1	5	5	6
35036	CIR	0	49	1	5	3	6
35060	CIR	0	58	1	5	3	6
35066	CIR	0	59	1	6	6	5
35074	CIR	0	48	2	6	5	6
35083	CIR	0	47	1	6	6	
35089	CIR	1	54	1	5	6	6
35094	CIR	0	47	2	5	5	5
35150	CIR	0	51	1	6	6	6
35153	CIR	1	55	1	6	5	
35165	CIR	1	53	1	6	6	6
35166	CIR	1	57	2	6	6	6
35181	CIR	0	45	1	6	6	5

				AVG	5.6	5.2	5.69
34926	FIB	0	47	1	3	3	3
34946	FIB	1	40	1	3	3	3
34958	FIB	0	47	1	4	3	3
34967	FIB	1	65	1	3	3	3
34973	FIB	1	53	1	4	4	2
34994	FIB	0	49	1	3	2	2
35008	FIB	0	55	1	4	4	4
35016	FIB	0	41	1	3		4
35033	FIB	0	51	1	4	4	3
35051	FIB	0	55	2	3	4	4
35062	FIB	0	44	1	3	2	2
35077	FIB	0	42	1	4	3	3
35080	FIB	0	47	2	3	1	2
35082	FIB	0	45	1	4	3	3
35098	FIB	1	50	2	3	4	4
				AVG	3.4	3.07	3
**Control Group**							
14758	CTRL	0	50	1			
13692	CTRL	0	51	1			
15962	CTRL	0	53	1			
20917	CTRL	0	46	1			
20919	CTRL	0	46	1			
13686	CTRL	0	46	1			
16020	CTRL	0	47	1			
13472	CTRL	0	47	1			
20913	CTRL	0	48	1			
20895	CTRL	0	56	2			
20912	CTRL	0	57	2			
20908	CTRL	1	54	1			
16970	CTRL	1	57	1			
14809	CTRL	1	57	1			
20887	CTRL	1	43	2			

		Mean Age	Male Gender	Race Caucasian	Race African American
Age	CTRL (n=15)	51	11	12	3
	Fibrosis (n=15)	49	11	12	3
	Cirrhosis (n=15)	52	11	12	3
Gender	Female	4			
	Male	11			
Race	White	12			
	Black	3			
